# Supplementary material for: Retail Food Environment around Schools in Barcelona by Neighborhood Socioeconomic Status: Implications for Local Food Policy
Source: Int J Environ Res Public Health. 2022 Dec 30;20(1):649. doi: 10.3390/ijerph20010649 (PMC9819657; doi:10.3390/ijerph20010649)
Supplement: Supplementary file 1 [file ijerph-20-00649-s001.zip › ijerph-2107145-supplementary.pdf]

Table S1. Healthy Food Availability Index (HFAI) using Nutrition Environment Measurement Survey in Store for Mediterranean contexts (NEMS-S-MED).

Item	Store type							
	Supermarkets[1] (*n*=18)	Convenience Stores[1] (*n*=36)	Fruit and Vegetables[1] (*n*=26)	Butcheries[1] (*n*=32)	Fishmongers [1] (*n*=16)	Bakeries[1] (*n*=23)	Other specialized food stores[1] (*n*=2)	p-Value[2]
**Availability**	26 (20-31)	7 (4-14)	8 (8-12)	3 (1-8)	4 (4-4)	1 (0-2)	1 (0-2)	0.0001
**Price**	6 (4-8)	0 (0-2)	0 (0-0)	0 (0-0)	0 (0-0)	0 (0-0)	0 (0-0)	0.0001
**Total Score**	31 (26-41)	7 (4-16)	8 (8-12)	3 (1-8)	4 (4-4)	1 (0-2)	1 (0-2)	0.0001

[1] Median (interquartile range), [2] p-values correspond to Kruskal-Wallis test.

Table S2. Association between the Healthy Food Availability Index (HFAI) of each food establishment and the socioeconomic status of schools at the neighborhood level.

	IRR[1]	95% CI	p-Value
**NSES**			
Low-SES	1.08	(0.96-1.23)	0.181
Middle	ref		
High-SES	1.67	(1.45-1.91)	0.000
**Population density (10^3 residents/km^2)**			
Medium	0.92	(0.81-1.04)	0.191
High	0.73	(0.64-0.84)	0.000

1IRR = incidence rate ratio, 95% CI = 95% confidence interquartile range, NSES: neighborhood socioeconomic status. All estimates are mutually adjusted for distance and all variables listed using Poisson regression.
